# Recommendations for nasotracheal tube insertion depths in neonates

**DOI:** 10.3389/fped.2022.990423

**Published:** 2022-08-22

**Authors:** Chinedu Ulrich Ebenebe, Kristina Schriever, Monika Wolf, Jochen Herrmann, Dominique Singer, Philipp Deindl

**Affiliations:** ^1^Division of Neonatology and Pediatric Intensive Care, Department of Pediatrics, University Medical Center Hamburg-Eppendorf, Hamburg, Germany; ^2^Section of Pediatric Radiology, Department of Interventional and Diagnostic Radiology and Nuclear Medicine, University Medical Center Hamburg-Eppendorf, Hamburg, Germany

**Keywords:** airway management, intubation, endotracheal, neonate, nasotracheal, preterm

## Abstract

**Background:**

Endotracheal tube (ETT) malposition is common in neonatal intubation. Recommendations for ETT insertion depths predominantly address orotracheal intubation. The aim of this study was to develop gestational age-, weight-, and length-based curve charts and tables for nasotracheal ETT insertion depth recommendations in neonates.

**Method:**

In this retrospective single-center study, the individual optimal ETT insertion depths in neonates were determined by evaluating postintubation radiographic images. Gestational age-, weight-, and length-based best-fit curves and tables were generated using regression analysis to calculate related ETT insertion depths. The insertion depths predicted by the models were compared with previously published recommendations.

**Results:**

We analyzed intubations of 178 neonates (gestational age range at intubation: 23.7–43.0 weeks). Applying sigmoidal logistic regression models, curves, and tables revealed *R*^2^ values between 0.766 and 0.837. The insertion depths predicted by the models revealed certain deviations when compared with four previously published recommendations for nasotracheal ETT depth estimation in neonates.

**Conclusion:**

The charts and tables developed in this study enable a fast and accurate determination of recommended nasotracheal ETT insertion depths in neonates.

## Introduction

Endotracheal intubation in neonates is a potentially life-saving procedure in the delivery room and the neonatal intensive care unit. Precise positioning of the endotracheal tube (ETT) is essential to reduce the incidence of complications, including atelectasis, barotrauma, pneumothorax, unplanned extubation, post-extubation stridor, and asymmetrical surfactant distribution (1).

Chest radiography is the gold standard for confirming the correct ETT position (2). Nevertheless, as ventilation must be initiated immediately after intubation and prompt bedside radiography is not consistently available, reliable methods to accurately predict the ETT insertion depth are essential. This is notably true for emergency neonatal transport, where an accurate assessment of effective respiratory support is paramount (3).

Numerous recommendations, most of which refer to orotracheal intubation, have been proposed to achieve correct tube placement within the trachea in neonates (4–7). However, several countries apply the nasotracheal intubation route as it offers advantages like easier fixation, reduced risk of unplanned extubation, and higher patient comfort (8). Also, commonly used formulae for ETT insertion depth induce a significant incidence of ETT malposition (9). The formula-based approach is possibly inaccurate because linear equations discount that fetal and neonatal growth does not entirely follow a linear relationship.

The aim of this study was to develop gestational age (GA)-, weight-, and length-based charts and tables to determine recommendations for correct nasotracheal ETT insertion depths based on a significant cohort of neonates.

## Method

### Study design and subjects

Intubated pediatric patients admitted to the neonatal intensive care unit of our tertiary perinatal center from January 2017 to March 2021 were retrospectively evaluated. Clinical patient data were obtained by reviewing the hospital’s healthcare information systems (Soarian^®^, Siemens Healthcare, Erlangen, Germany; ICM^®^, Draeger, Luebeck, Germany). Extracted information included sex, GA, weight, length, and ETT insertion depth.

Intubation was performed with uncuffed tubes with the distal 5 mm beveled by 38° (Vygon, Aachen, Germany). Routine postintubation chest radiography was conducted in an anterior-posterior position, with a focus-to-film distance of 100 cm, and the child’s head placed in the midline position. Radiographs were taken with the mobile digital radiography systems Samsung GM60 (January 2017 to January 2018) and Samsung GM85 (February 2018 to March 2021) in combination with the digital flat panel X-ray detector S3025-W (Samsung Electronics GmbH, Schwalbach, Germany). Two different investigators measured the tip-to-carina distance independently on the radiograph images. Patients were excluded if the radiograph’s quality was too poor to identify ETT tip and carina. Interobserver discrepancies were resolved by re-evaluation.

The patients were allocated into predefined categories for GA, weight, and length (Table 1). A distinct category-dependent ETT tip-to-carina distance was determined, with a target ETT tip location at the mid-trachea position (10). The optimal ETT tip-to-carina distance for each patient was calculated based on data from Szpinda et al. and Cerone et al., who measured tracheal lengths during different gestational stages (Table 1) (11, 12). The optimal ETT depth for each patient was determined by adjusting the recorded ETT depth by the distance between the recorded and the defined optimal ETT tip position. Patients were excluded if the radiograph quality was too poor to identify the exact tip position. Also, in patients who underwent multiple intubations, only the first episode was included in the analysis to exclude cluster effects.

**TABLE 1:** Gestational age-, weight-, and length-based categories with the corresponding optimal ETT tip-to-carina distance.

Category	Gestational age (weeks)	Weight (g)	Length (cm)	Average tracheal length (cm) (11, 12)	Optimal ETT tip-to-carina distance (cm)
1	≤24	≤700	≤32	2.8	1.4
2	>24–30	>700–1,400	>32–40	3.2	1.6
3	>30–36	>1,400–2,800	>40–48	4.0	2.0
4	>36	>2,800	>48	5.0	2.5

Ethical approval for this study (WF-x159/20) was provided by the ethical committee of the local medical chamber.

### Statistical analysis

Statistical analysis was performed using SPPS, version 27 (IBM, Armonk, NY, United States) and GraphPad Prism 9 (GraphPad, La Jolla, CA, United States). Data on neonatal demographics were expressed as the median and range for continuous variables and as counts and percentages for categorical variables.

The individual optimal ETT insertion depth was plotted against GA, weight, and length for all neonates. The best-fit curves for proposed ETT insertion depths were calculated using linear and non-linear regression models. The 95% prediction bands enclosing the area that included 95% of future data points were generated. *R*^2^ values were calculated to determine the goodness-of-fit for each regression model.

The target ETT insertion depths based on our data and previously published recommendations were plotted for comparison.

## Results

### Demographic characteristics

Study criteria were met by 178 patients (Supplementary Figure 1), of which 81 (45.5%) were neonates ≤32 weeks of GA, were analyzed. The basic demographic characteristics are shown in Table 2.

**TABLE 2:** Demographic characteristics of 178 included neonates at time of intubation.

Characteristics	Values
Female, *n* (%)	85 (47.8)
Age (days)	0 (0–27)
Gestational age (weeks)	34.6 (23.7–43.0)
Weight (g)	2,000 (440–4,900)
Length (cm)	44.5 (28–59)

Values are given as median (range) unless stated otherwise.

### Generating best-fit curves and tables for proposed endotracheal tube insertion depth

**FIGURE 1:**
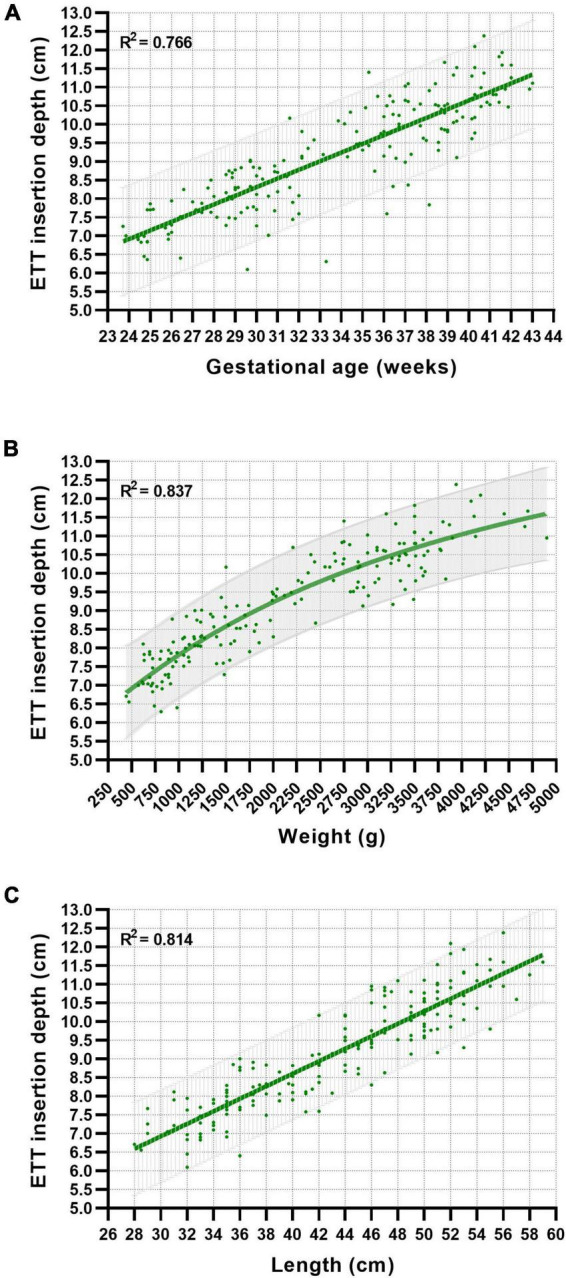
Relationship between gestational age **(A)**, weight **(B)**, length **(C)**, and proposed nasotracheal ETT insertion depths in neonates. Best-fit curves and 95% prediction bands were generated using linear and non-linear regression models.

The calculated optimal ETT insertion depth for each neonate was plotted against GA, weight, and length with the corresponding best-fit curves and 95% prediction bands (Figure 1). Best-fit curves with the highest accuracy were generated using linear regression for GA and length and a sigmoidal four-parameter logistic regression model for weight. The *R*^2^ values revealed a more accurate fit of the regression models for weight and length-related curves than the GA-related curve (Figure 1). Overall comparison between male and female neonates revealed no significant differences in the proposed ETT insertion depth.

Values for the recommendation of GA-, weight-, and length-based ETT insertion depths interpolated from the best-fit curves are presented in Supplementary Table 1.

### Comparison of the proposed endotracheal tube insertion depth curve charts with historical formulae

**FIGURE 2:**
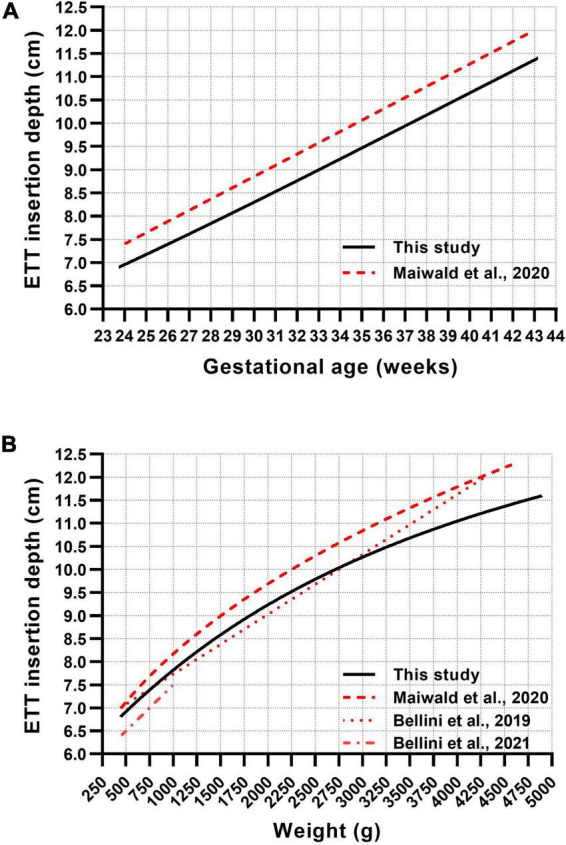
Comparison between published gestational age-based **(A)** and weight-based **(B)** formulae and our generated curve charts for the recommendation of nasotracheal ETT insertion depths in neonates.

Four published recommendations for nasotracheal ETT depth estimation in neonates were compared with our curves (Figure 2). The GA-based reference by Maiwald et al. runs parallel to our line, differing by approximately 5 mm more insertion depth (Figure 2A) (13). In the weight-based recommendation, all curves lie relatively close in very low birth weight neonates and continuously diverge in bigger infants (Figure 2B). The linear weight-based curve by Bellini et al. runs quite close to our curve in neonates less than 3,000 g but increasingly deviates from our recommendation in bigger infants (Figure 2B) (14). The recommendation by Bellini et al. for extremely low birth weight (ELBW) infants (450–1,000 g) differs by about 3 mm less insertion depth (15).

## Discussion

Endotracheal tube malposition after intubation remains a frequent event in neonates. Several formulae have been proposed to estimate the optimal ETT insertion depth in neonates. However, these are not always accurate, particularly when using weight-derived formulae at the extremes of low GA or in small for GA infants (16). Also, data on ETT insertion depths referring to the nasotracheal route hardly exist. Therefore, we aimed to generate recommendations for nasotracheal ETT insertion depths based on an extensive data set of neonates of different GA groups calculated using linear and logistic regression models.

Consistent with the findings of previous studies, the relationship to the optimal ETT depth was linear for GA and body length and non-linear for body weight (Figure 1) (13, 17). The goodness-of-fit of the generated best-fit curves, represented by the *R*^2^ value, varied depending on the associated parameter. The best accuracy was observed in the weight-based curve charts (*R*^2^ = 0.837) compared to length-based (*R*^2^ = 0.814) or GA-based (*R*^2^ = 0.766) estimations. This is consistent with the findings of Maiwald et al. demonstrating a goodness-of-fit for the weight- and GA-based regression models (0.85 and 0.79, respectively) similar to our study.

We identified four published recommendations for nasotracheal ETT insertion depth in neonates (13–15). The recommendations by Maiwald et al. resemble our findings, however, indicating 5 mm more depth in the GA-based curves and 2–8 mm in the weight-based curves (Figures 2A,B) (13). The correspondence was less in the higher weight ranges, where certain deviations are more likely tolerable in the clinical context. The variance is presumably due to the differing definitions of the optimal ETT position. While we calculated the mid-trachea position using historical data from the measured trachea, Maiwald et al. defined the optimal ETT position at the middle of vertebra T2, representing the lower zone of a commonly applied target range of T1–T2 (18). This could also explain why the formula for ELBW infants by Bellini et al. (15), defining the optimal tip position at T1–T2, differs by approximately 3 mm less insertion depth compared to our recommendations and 6–7 mm compared to Maiwald et al., which may be of relevance in this weight group. Due to its linear character, the weight-based recommendation by Bellini et al. corresponds quite well only in neonates less than 3,000 g but increasingly deviates from our recommendation in bigger infants (Figure 2B) (14).

So far, no length-based recommendations for nasotracheal ETT depths have been published. However, length-related guidance demonstrated a similar accuracy as weight and may be especially useful in the delivery room setting, where the body length can be easily determined.

The charts and tables presented in this study enable a rapid recommendation of ETT insertion depths and can be a valuable initial guiding tool for neonatal intubation. However, the accuracy and performance of our recommendations require prospective validation.

This study has some limitations. Due to the study’s retrospective design, we estimated the optimal ETT depths using indirect methods. Our clinical routine includes conducting chest X-rays with the head in a neutral position. However, we could not verify the degree of the head-neck flexion in this retrospective study.

## Conclusion

We present accurate recommendations for nasotracheal ETT insertion depths for neonates as charts and tables based on a large data set.

## Data availability statement

The raw data supporting the conclusions of this article will be made available by the authors, without undue reservation.

## Ethics statement

The studies involving human participants were reviewed and approved by the Ärztekammer Hamburg. Written informed consent from the participants’ legal guardian/next of kin was not required to participate in this study in accordance with the national legislation and the institutional requirements.

## Author contributions

CE: conceptualization, methodology, formal analysis, writing—original draft, and project administration. KS: methodology, formal analysis, and investigation. MW, JH, and DS: visualization and writing—review and editing. PD: methodology, formal analysis, writing—review and editing, supervision, and project administration. All authors contributed to the article and approved the submitted version.

## Supplementary material

Supplementary Figure 1 Flow diagram of enrolment.
