# Supplementary material for: Recommendations for nasotracheal tube insertion depths in neonates
Source: Front Pediatr. 2022 Aug 22;10:990423. doi: 10.3389/fped.2022.990423 (PMC9441670; doi:10.3389/fped.2022.990423)
Supplement: Supplementary file 1 [file Table_1.DOCX]

**Supplementary Material - Table 1.** Data table with recommended ETT insertion depths for nasotracheal intubation in relation to gestational age, weight, and length interpolated from the corresponding best-fit curves

| ETT depth (cm) | GA (weeks) | Weight (g) | Length (cm) |
| --- | --- | --- | --- |
| 6.5 | <23.1 | <415 | <24.0 |
| 7.0 | 23.1–25.3 | 415–675 | 24.0–31.2 |
| 7.5 | 25.4–27.5 | 676–959 | 31.3–35.4 |
| 8.0 | 27.6–29.7 | 960–1272 | 35.5–38.5 |
| 8.5 | 29.8–31.9 | 1273–1621 | 38.6–41.3 |
| 9.0 | 32.0–34.0 | 1622–2015 | 41.4–43.9 |
| 9.5 | 34.1–36.1 | 2016–2464 | 44.0–46.6 |
| 10.0 | 36.2–38.3 | 2465–2982 | 46.7–49.4 |
| 10.5 | 38.4–40.4 | 2983–3588 | 49.5–52.5 |
| 11.0 | 40.5–42.5 | 3589–4309 | 52.6–56.3 |
| 11.5 | 42.6–44.6 | 4310–5183 | 56.4–61.5 |

Abbreviations: ETT, endotracheal tube; GA gestational age.
