# Supplementary figures and images for: Recommendations for nasotracheal tube insertion depths in neonates
Source: Front Pediatr. 2022 Aug 22;10:990423. doi: 10.3389/fped.2022.990423 (PMC9441670; doi:10.3389/fped.2022.990423)

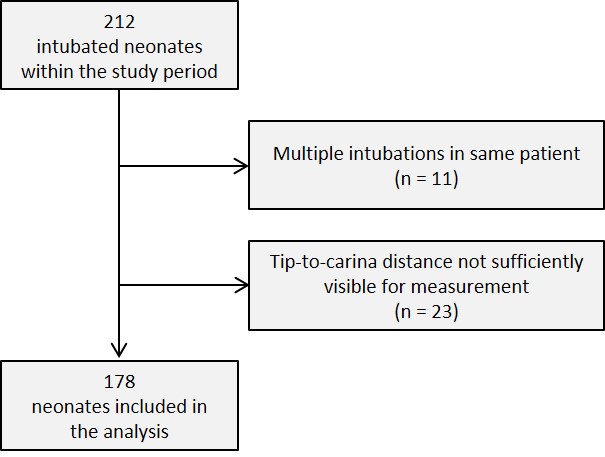

Supplement: Supplementary Figure 1 — Flow diagram of enrolment. [file Image_1.JPEG]
